# Incomplete and late recovery of sudden olfactory dysfunction in COVID-19^☆^^☆☆^

**DOI:** 10.1016/j.bjorl.2020.05.001

**Published:** 2020-05-25

**Authors:** Eduardo Macoto Kosugi, Joel Lavinsky, Fabrizio Ricci Romano, Marco Aurélio Fornazieri, Gabriela Ricci Luz-Matsumoto, Marcus Miranda Lessa, Otávio Bejzman Piltcher, Geraldo Druck Sant’Anna

**Affiliations:** a Academia Brasileira de Rinologia (ABR), São Paulo, SP, Brazil; b Associação Brasileira de Otorrinolaringologia e Cirurgia Cérvico-Facial (ABORL-CCF), São Paulo, SP, Brazil

**Keywords:** COVID-19, Anosmia, Olfactory disorders, COVID-19, Anosmia, Transtornos do olfato

## Abstract

**Introduction:**

Sudden olfactory dysfunction is a new symptom related to COVID-19, with little data on its duration or recovery rate.

**Objective:**

To characterize patients with sudden olfactory dysfunction during the COVID-19 pandemic, especially their recovery data.

**Methods:**

An online survey was conducted by the Brazilian Society of Otorhinolaryngology and Cervico-Facial Surgery, and Brazilian Academy of Rhinology, including doctors who assessed sudden olfactory dysfunction patients starting after February 1st, 2020. Participants were posteriorly asked by e-mail to verify data on the recovery of sudden olfactory loss and test for COVID-19 at the end of the data collection period.

**Results:**

253 sudden olfactory dysfunction patients were included, of which 59.1% were females with median age of 36 years, with a median follow-up period of 31 days. 183 patients (72.3%) had been tested for COVID-19, and of those 145 (79.2%) tested positive. Patients that tested positive for COVID-19 more frequently showed non-specific inflammatory symptoms (89.7% vs. 73.7%; *p* = 0.02), a lower rate of total recovery of sudden olfactory dysfunction (52.6% vs. 70.3%; *p* = 0.05) and a longer duration to achieve total recovery (15 days vs. 10 days; *p* = 0.0006) than the ones who tested negative for COVID-19. Considering only positive-COVID-19 patients, individuals with sudden hyposmia completely recovered more often than the ones with sudden anosmia (68.4% vs. 50.0%; *p* = 0.04).

**Conclusion:**

Positive-COVID-19 patients with sudden olfactory dysfunction showed lower total recovery rate and longer duration than negative-COVID-19 patients. Additionally, total recovery was seen more frequently in positive-COVID-19 patients with sudden hyposmia than the ones with sudden anosmia.

## Resumo

**Introdução:**

A perda súbita do olfato é um novo sintoma relacionado à Covid-19, porém com poucos dados sobre sua duração ou resolução.

**Objetivo:**

Caracterizar pacientes que apresentaram perda súbita do olfato durante a pandemia da COVID-19 e em especial a sua recuperação.

**Método:**

Pesquisa *online* desenvolvida pela Associação Brasileira de Otorrinolaringologia e Cirurgia Cérvico-Facial e Academia Brasileira de Rinologia direcionado aos médicos que atenderam pacientes com perda súbita do olfato com início após 1° de fevereiro de 2020. Os participantes foram questionados posteriormente por e-mail, para verificar os dados sobre a recuperação da perda súbita do olfato e teste para COVID-19, no final do período de coleta de dados.

**Resultados:**

Foram incluídos 253 pacientes com perda súbita de olfato, 59,1% mulheres e idade mediana de 36 anos, acompanhados por 31 dias (mediana). Testagem para COVID-19 foi feita em 183 (72,3%) pacientes, 145 (79,2%) positivos e 38 (20,8%) negativos. COVID-19 positivos apresentaram sintomas inflamatórios inespecíficos mais frequentemente (89,7% *vs.* 73,7%; *p* = 0,02); menor taxa de recuperação total da perda súbita do olfato (52,6% *vs.* 70,3%; *p* = 0,05) e maior tempo para atingir a recuperação total (15 dias *vs.* 10 dias; *p* = 0,0006) comparados aos COVID-19 negativos. Considerando somente COVID-19 positivos, hiposmia súbita apresentou melhora total mais frequentemente que anosmia súbita (68,4% *vs.* 50,0%; *p* = 0,04).

**Conclusão:**

A perda súbita do olfato em pacientes COVID-19 positivos apresentou menor taxa de recuperação total e duração mais prolongada do que em COVID-19 negativos. E a hiposmia súbita apresentou recuperação total mais frequentemente que a anosmia súbita em COVID-19 positivos.

## Introduction

Human Coronaviruses (HCoVs) were first identified in the nasal cavities of patients with the common cold in the 1960s,^1^ being responsible for 10–15% of these cases, second only to rhinoviruses.^2^ Although most cases of HCoV infection show symptoms compatible with the common cold or mild flu-like syndromes, the lower respiratory tract can be severely affected, as in outbreaks caused by the SARS-CoV, MERS-CoV species, and now with the new coronavirus (SARS-COV-2), which causes the COVID-19 disease.^1^ In China, the analysis of 72,314 cases up to February 11, 2020 showed that 14% of patients with COVID-19 had severe disease and 5% were critically ill, leading to a case fatality rate of 2.3%.^3^ In Brazil, at the beginning of May 2020, there were already more than 145,000 confirmed cases, with a case fatality rate reaching 6.8%.^4^

In addition to respiratory symptoms, an unusual finding started to be noticed in patients with COVID-19: sudden anosmia. A study focusing on neurological alterations showed only 5.1% of changes in smell in patients hospitalized with COVID-19 in Wuhan, China,^5^ compatible with a prevalence of 5.8% of anosmia in population studies.^6^ However, in Europe, 85.6% of patients with mild to moderate COVID-19 had a sudden change in olfaction, with 79.6% anosmia and 20.4% hyposmia.^7^ This finding did not seem to follow the usual pattern of post-viral olfaction alterations: in the USA, a study comparing patients with flu-like symptoms showed a 16% prevalence of post-viral olfaction alteration, which increased to 68% in positive-COVID-19 patients,^8^ similar to the European and in disagreement with the Chinese data. Interestingly, in the first study using olfactory tests in these patients, carried out in Iran, the prevalence of olfactory dysfunction assessed in those infected with SARS-CoV-2 reached 98%.^9^

The reports of sudden anosmia by COVID-19 led the Brazilian Academy of Rhinology (ABR, *Academia Brasileira de Rinologia*) and the Brazilian Association of Otorhinolaryngology and Cervical-Facial Surgery (ABORL-CCF, *Associação Brasileira de Otorrinolaringologia e Cirurgia Cérvico-Facial*) to issue the “4th Guidance Note to Otorhinolaryngologists in relation to the disease caused by the New Coronavirus (COVID-19)”, on March 22, 2020, advising that the presence of sudden anosmia (with or without ageusia and without concomitant nasal obstruction) could suggest the presence of COVID-19 in this scenario of pandemic and sustained transmission of the SARS-CoV-2 virus.^10^

Considering this new clinical presentation of COVID-19 and the possible variation in susceptibility to anosmia caused by the SARS-CoV-2 virus in different populations, it is necessary to assess the characteristics of sudden olfactory dysfunction in this context of the COVID-19 pandemic in the Brazilian population, as there is, to date, no Brazilian data on this topic. Moreover, little is known about the evolution of sudden loss of olfaction related to COVID-19. Thus, the aim of this study was to characterize patients who experienced sudden olfactory dysfunction during the COVID-19 pandemic, and in particular, their recovery.

## Methods

An online survey was developed by ABORL-CCF and ABR to be filled out by physicians who treated patients with sudden olfactory dysfunction starting on February 1, 2020 in Brazil in this context of the COVID-19 pandemic. The research investigated the epidemiological profile of patients with sudden anosmia or hyposmia, associated symptoms, comorbidities, treatment used, recovery from sudden anosmia or hyposmia, if there was a test to confirm COVID-19, and the result of this test. The survey was distributed digitally by ABORL-CCF via its website (www.aborlccf.org.br), Whatsapp® and Instagram®. Data were collected between March 25 and April 30, 2020. The participating physicians were asked for authorization to use the included data. Data on the recovery from sudden anosmia or hyposmia and test for COVID-19 were confirmed via e-mail, and information on the time for total recovery from anosmia or hyposmia and how long the patient was followed by the doctor were also collected.

Symptom prevalence rates were described in percentages, whereas continuous variables, such as duration of complaints and age, were described in medians and Interquartile Range (IQR). Proportions were compared using Fisher's exact or Chi-square test. The difference in age distribution, time to total recovery and duration of patient follow-up were calculated using the Mann–Whitney *U* test due to the non-normal distribution of data calculated by the Kolmogorov-Smirnov test. The level of significance was set at 5% and the tests used were two-tailed.

## Results

A total of 253 patients was included, treated in all regions of Brazil, distributed as follows: 142 (56.1%) in the Southeast, 59 (23.3%) in the Northeast, 32 (12.6%) in the South, 10 (4.0%) in the North and 10 (4.0%) in the Midwest. There was a predominance of women (149 patients - 58.9%), and the median age was 36 years (IQR 30–44 years).

Most patients had sudden anosmia (212 patients - 83.8%) instead of sudden hyposmia, and in most cases (196 patients - 77.5%), loss of smell was accompanied by nonspecific inflammatory symptoms (coughing, fever, headache, fatigue/malaise, myalgia/arthralgia and/or anorexia). Nasal symptoms (nasal obstruction, sneezing, coryza, purulent rhinorrhea, nasal pruritus and/or nasal burning) were reported by 111 patients (43.9%) and sore throat by 16 (6.3%). Only 36.4% of the patients reported comorbidities, with rhinitis being the most frequent one (53 patients – 20.9%), followed by asthma and systemic arterial hypertension (SAH), with 13 patients each (5.1%). Chronic rhinosinusitis was reported by 7 patients (2.8%).

The treatment was expectant in most cases (124 patients - 49.0%). When opting for some treatment, nasal saline irrigation (NSI) was the one most frequently chosen (66 patients – 26.1%); followed by analgesics/antipyretics in 30 patients (11.9%); topical intranasal corticosteroids (INCS) in 28 patients (11.1%); antibiotics in 22 patients (8.7%); oral corticosteroids (oral CS) in 12 patients (4.7%); hydroxychloroquine in 6 patients (2.4%); oseltamivir in 5 patients (2.0%) and olfactory training in 5 patients (2.0%).

The information on the recovery of the sudden olfactory dysfunction was obtained from 227 records (89.7%): 121 patients with full recovery (53.3%), 76 with partial recovery (33.5%) and 30 with no recovery (13.2%). When there was full recovery, the time to achieve this result showed a median of 12.5 days (IQR 9.25–20.75 days). Additionally, the time of follow-up lasted a median of 31 days (IQR 10.5–39 days).

Most patients (183–72.3%) were tested for confirmation of COVID-19, with 145 (79.2%) positive and 38 (20.8%) negative results. The untested patients were younger (median of 34 years vs. 36 years; *p* = 0.05), with more isolated anosmia (25.7% vs. 7.1%; *p* = 0.0001); fewer nonspecific inflammatory symptoms (54.3% vs. 86.3%; *p* < 0.0001) and shorter time of follow-up (median 15 days vs. 31.5 days; *p* = 0.007) than the tested patients.

Sub-analyses were performed between positive- and negative-COVID-19 patients. There were no differences regarding epidemiological characteristics between positive- and negative-COVID-19 patients, as shown in Table 1.

**Table 1:** General characteristics of patients tested for COVID-19.

Characteristics of tested patients (*n* = 183)		COVID-19 test				*p*-Value
		Positive (*n* = 145)		Negative (*n* = 38)		
Age (years)	Med and IQR	36	31–44	35.5	30.25–45.25	0.60
Follow-up (days)	Med and IQR	31	12–39	35.5	17–41	0.37
Female gender	*n* and %	77	53.1	26	68.4	0.10
Acute anosmia	*n* and %	126	86.9	31	81.6	0.44
Acute hyposmia	*n* and %	19	13.1	7	18.4	0.44
Comorbidities	*n* and %	47	32.4	15	39.5	0.44
Allergic rhinitis	*n* and %	22	15.2	8	21.1	0.46
Asthma	*n* and %	7	4.8	0	0.0	0.35

*n*, number; Med, median; IQR, interquartile range; %, percentage.

Positive-COVID-19 patients more commonly had nonspecific inflammatory symptoms (coughing, fever, headache, fatigue/malaise, myalgia/arthralgia and/or anorexia) than negative-COVID-19 ones (89.7% vs. 73.7%; *p* = 0.02*). There were no statistically significant differences between the two groups for the other symptoms, as shown in Table 2. There were also no differences regarding the patterns of treatment used in the two groups of patients.

**Table 2:** Symptoms of patients tested for COVID-19.

Symptoms of tested patients (*n* = 183)	COVID-19 test				*p*-Value
	Positive (*n* = 145)		Negative (*n* = 38)		
	*n*	%	*n*	%	
None	8	5.5	5	13.1	0.15
Nonspecific symptoms	130	89.7	28	73.7	0.02^a^
Coughing	83	57.2	18	47.3	0.36
Headache	76	52.4	20	52.6	1.00
Fever	72	49.7	16	42.1	0.47
Myalgia/arthralgia	37	25.5	8	21.0	0.68
Fatigue	20	13.7	7	18.4	0.14
Anorexia	4	2.7	2	5.2	0.61
Nasal symptoms	62	42.7	15	39.4	0.85
Nasal obstruction	40	27.5	8	21.0	0.54
Sneezing	18	12.4	4	10.5	1.00
Coryza	31	21.3	8	21.0	1.00
Rhinorrhea	2	1.3	1	2.6	0.51
Nasal pruritus	6	4.1	2	5.2	0.67
URTIs (nose and/or throat)	66	45.5	16	42.1	0.86
Sore throat	10	6.9	4	10.5	0.56
Others					
Dyspnea	7	4.8	2	5.2	1.00
Diarrhea	10	6.9	1	2.6	0.46
Ageusia	6	4.1	2	5.2	0.67

*n*, number; %, percentage.

a Statistical significance.

**Figure 1:**
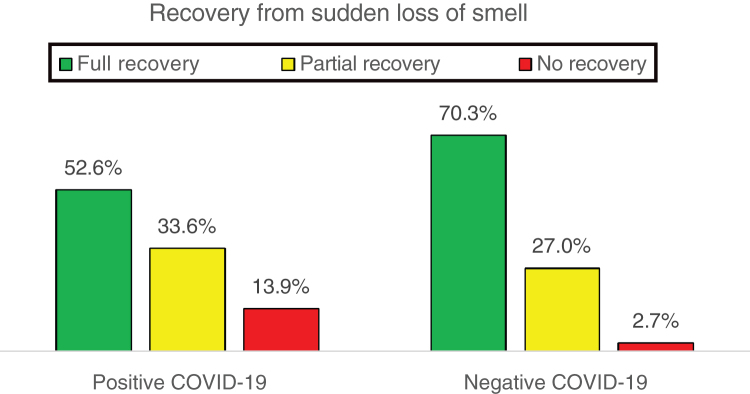
Recovery from sudden loss of smell between positive- and negative-COVID-19 individuals (*p* = 0.05).

Sudden olfactory dysfunction in the positive-COVID-19 patients showed a lower full recovery rate (Fig. 1) and a longer duration than in the negative-COVID-19 ones (Table 3). Full recovery from sudden olfactory dysfunction was less frequent in the positive-COVID-19 than in the negative-COVID-19 patients (52.6% vs. 70.3%; *p* = 0.05); and the time to achieve full recovery from the sudden olfactory dysfunction was longer in the positive-COVID-19 than in the negative-COVID-19 patients (median of 15 days vs. 10 days; *p* = 0.0006), although the time of follow-up was not different between positive- and negative-COVID-19 patients (median 31 days vs. 35.5 days; *p* = 0.37).

**Table 3:** Recovery from loss of olfaction in patients tested for COVID-19.

Recovery from sudden olfactory dysfunction (*n* = 174)		COVID-19 test				*p*-Value
		Positive (*n* = 137)		Negative (*n* = 37)		
Full recovery	*n* and %	72	52.6	26	70.3	0.05^a^
Partial recovery	*n* and %	46	33.6	10	27.0	
No recovery	*n* and %	19	13.9	1	2.7	
Time to full recovery	Med and IQR	15	10–21	10	8–12.75	0.0006^a^

*n*, number; %, percentage; Med, median; IQR, interquartile range.

a Statistical significance.

When analyzing only positive-COVID-19 patients, it was observed that those with hyposmia recovered more easily than the ones with anosmia (Table 4); there was no difference in the full recovery from sudden olfactory dysfunction due to the different treatments used; and that there was no association between full recovery from sudden olfactory dysfunction due to the presence or absence of other symptoms.

**Table 4:** Recovery from acute anosmia and hyposmia in positive-COVID-19 patients.

Recovery from sudden olfactory dysfunction (*n* = 137)		Positive-COVID-19				*p*-Value
		Anosmia (*n* = 118)		Hyposmia (*n* = 19)		
Full recovery	*n* and %	59	50.0	13	68.4	
Partial recovery	*n* and %	44	37.3	2	10.5	0.04^a^
No recovery	*n* and %	15	12.7	4	21.1	
Time to full recovery	Med and IQR	15.5	10.75–23	11	9–16	0.10

*n*, number; %, percentage; Med, median; IQR, interquartile range.

a Statistical significance.

## Discussion

This is the first Brazilian study to assess sudden olfactory dysfunction in the context of the COVID-19 pandemic. The most relevant finding in the present study was the demonstration that the sudden olfactory dysfunction associated with COVID-19 had a lower rate of full recovery and a longer duration than in negative-COVID-19 patients. Only half of the positive-COVID-19 patients fully recovered olfaction, compared to 70.3% of the negative-COVID-19 ones (*p* = 0.05). Considering only those who fully recovered the olfaction, it took positive-COVID-19 patients 15 days (median) to fully recover their sense of smell, 5 days longer than negative-COVID-19 ones (*p* = 0.0006). The second relevant point is that the full recovery from sudden hyposmia in positive-COVID-19 patients occurred more frequently than that from sudden anosmia (*p* = 0.04).

Women have complained more about loss of olfaction during the COVID-19 pandemic, representing up to three quarters of the cases,^11^ which, however, is reduced to two-thirds when there is diagnostic confirmation of COVID-19.^7^ The present research included a total of 59.1% of women with olfactory dysfunction, being 53.1% when positive-COVID-19 cases were confirmed. However, it is interesting to mention that population studies tend to show a higher prevalence of olfactory dysfunction in men.^6^ This skewed distribution of loss of smell in COVID-19 may be due to the decreased capacity of men to perceive the olfactory dysfunction, or even women's greater concern for their health, or an actual selectivity of SARS-CoV-2 for the female gender.

Another discrepant point between the olfactory dysfunction related to COVID-19 and that found in the general population is the anosmia/hyposmia ratio. The prevalence of hyposmia is usually more than two-fold that of anosmia,^6^ but the studies by Hopkins et al.^11^ and Lechien et al.^7^ showed that 74.4 and 76.9% of the olfactory dysfunction cases related to COVID-19 were those of anosmia, not hyposmia. In agreement with that, 83.4% of olfaction complaints in the present study were of anosmia, reaching 86.9% in positive-COVID-19 patients.

Although HCoVs are the second most frequent cause of acute nasopharyngitis,^2^ SARS-CoV-2 does not seem to predominantly promote nasal or pharyngeal conditions,^7^, ^8^, ^11^, ^12^, ^13^ hence, the recommendation made by the 4th Guidance Note of ABR/ABORL-CCF was to always suspect COVID-19 in the current scenario, in cases of sudden anosmia that was not accompanied by nasal obstruction.^10^ However, among the positive-COVID-19 patients in this study, 27.5% had nasal obstruction concomitantly. Therefore, we should not exclude the possibility of COVID-19 just because the patient has upper airway symptoms. Sore throat was uncommon in the present study (6.9% vs. 10.5%; *p* = 0.56), according to the findings of Yan et al.^8^ who showed that sore throat was independently associated with negative-COVID-19 patients, with these patients being 4 to 5 times more likely to report sore throat as a symptom than the positive ones.

Nonspecific inflammatory symptoms such as coughing, headache, fever, myalgia/arthralgia and fatigue are usually the most prevalent ones in COVID-19.^7^, ^8^, ^11^, ^12^, ^13^ In the present study, positive-COVID-19 patients with sudden olfactory dysfunction had more general inflammatory symptoms (with coughing, headache and fever being the most common ones) than negative-COVID-19 patients (89.7% vs. 73.7%; *p* = 0.02*), corroborating the supposition that SARS-CoV-2 causes predominantly nonspecific inflammatory symptoms.

Sudden olfactory dysfunction alone (with no other symptoms) is not the norm. Hopkins et al.^11^ showed that only 16% of patients had sudden olfactory dysfunction without other symptoms, consistent with our global finding of 12.1% of isolated anosmia/hyposmia in the context of the pandemic. It is worth mentioning that, among our positive-COVID-19 patients, only 5.5% had isolated sudden olfactory dysfunction.

Dyspnea was not a frequent finding in the present study, which may represent the inclusion of a profile of patients with mild to moderate disease. Moreover, the presence of sudden anosmia seems to be related to the milder forms of COVID-19, as shown by Yan et al.^13^ Alterations in taste were not objectively assessed in this research, which may justify its low prevalence when compared to studies that showed high rates of ageusia associated with sudden anosmia in COVID-19 cases.^8^, ^13^

Hopkins et al., in a study with a similar design, showed that 80.1% of the patients with sudden olfactory dysfunction had some degree of olfactory recovery one week after the research, thus showing that it is likely that the olfaction will recover well in this COVID-19 pandemic scenario. However, full recovery from sudden olfactory dysfunction was reported by only 11.5% of the patients, and only 5.3% of patients in the study were tested for COVID-19, making it difficult to extrapolate the data.^14^ Our global data also showed high rates of some reported degree of olfactory recovery (86.8%) over a 31-day follow-up period (median), but this does not mean full recovery from sudden olfactory dysfunction, that is, return to the levels before the sudden loss of smell, which is desired by patients and doctors. Considering that 183 patients (72.3%) were tested for COVID-19, it was possible to compare the result of sudden loss of smell between positive- and negative-COVID-19 patients. Positive-COVID-19 patients had a lower full recovery rate from sudden olfactory dysfunction than negative-COVID-19 ones (52.6% vs. 70.3%; *p* = 0.05*). Moreover, positive-COVID-19 patients who managed to attain full recovery took 5 days longer than negative ones (median 15 days vs. 10 days; *p* = 0.0006*). Therefore, our data showed that SARS-CoV-2 infection caused a type of sudden olfactory dysfunction that was more difficult to resolve than sudden anosmia/hyposmia in negative-COVID-19 patients (which may possibly represent other post-viral olfaction losses, since in the present sample, only 13.2% of negative-COVID-19 patients did not have other associated infection symptoms). There are no data on the recovery of post-viral sudden olfactory dysfunction in the acute phase; therefore, the present study may bring new data in this sense as well. The present study also showed that sudden hyposmia in the positive-COVID-19 patients had a better evolution than those with sudden anosmia, also an unprecedented finding in the literature. Testing for COVID-19 is not the norm in outpatients in most countries, so much so that previous studies of sudden olfactory dysfunction in COVID-19 with questionnaires showed low rates of tested patients included in the studies, ranging from 3.3% to 5.3%.^11^, ^14^

The present study involved 72.3% of patients tested for COVID-19. The untested patients had a more benign profile (younger age and fewer associated symptoms), which probably led to the shorter time of follow-up and possibly to the lack of testing, due to the difficulties experienced in the pandemic for this purpose.

The positive points of the present study were the inclusion of a large sample of patients with sudden olfactory dysfunction, with a large percentage of patients being tested for COVID-19, and with a median follow-up of one month to assess the recovery from sudden olfaction loss. The negative point lies in the purely subjective analysis of sudden anosmia or hyposmia, which is not as reliable as the psychophysical tests of olfaction evaluation.^15^ On the other hand, these more sensitive tests can indicate changes in olfaction in more than 50% of individuals without olfactory complaints^16^ and, ultimately, it is the subjective perception of one's sense of smell that matters to the patient.

## Conclusion

The sudden olfactory dysfunction in positive-COVID-19 patients showed a lower total recovery rate and longer duration than in negative-COVID-19 patients. Only half of the positive-COVID-19 patients fully recovered their sense of smell, with a median time of 15 days to attain recovery. Additionally, positive-COVID-19 patients with sudden hyposmia recovered more frequently than the ones with sudden anosmia.

## Conflicts of interest

The authors declare no conflicts of interest.
